# Sakshat Labs: India's Virtual Proteomics Initiative

**DOI:** 10.1371/journal.pbio.1001353

**Published:** 2012-07-10

**Authors:** Sandipan Ray, Nicole R. Koshy, Shyam Diwakar, Bipin Nair, Sanjeeva Srivastava

**Affiliations:** 1 Wadhwani Research Center for Biosciences and Bioengineering, Department of Biosciences and Bioengineering, Indian Institute of Technology Bombay, Powai, Mumbai, India; 2 Amrita School of Biotechnology, Amrita Vishwa Vidyapeetham, Amritapuri, Kollam, Kerala, India

## Abstract

The first Virtual Proteomics Lab of India has been developed at the IIT Bombay as a part of the “Sakshat” Lab Project, established to develop openly accessible, high-quality educational materials on science and technology.

## Introduction

Thanks to the radical progress of information technology (IT), e-learning and open-learning resources are accessible to anyone, anywhere through the Internet. True open-learning resources can be freely accessed, reused, modified, and shared without restriction. Over the last decade, Open Course Ware (OCW) resources, originally initiated by MIT and the Hewlett Foundation, accelerated the spread of knowledge outside of the United States and Europe for global distribution, and were introduced in many Asian countries, including China and Japan [1]–[4]. However, high-quality educational resources for biotechnology and other life sciences disciplines are lacking [5]. The need for these open-learning programs in the broad field of biotechnology is 2-fold. Cutting-edge scientific research requires access to the latest techniques, which must be constantly refined to achieve greater accuracy in results; however, keeping pace with these techniques is no small feat. Economic crunches and perpetually limited educational resources make it difficult for most of the institutes to afford even the most basic laboratory set-ups [6]. Even well-funded institutions must limit their infrastructure to what they require for research purposes, and access to advanced labs is most often restricted to authorized research personnel.

Virtual labs, where simulators are used to create interactive tutorials that provide students a visual demonstration of techniques, may circumvent many of these roadblocks. While in conventional labs, students have only a short time to assimilate lessons, virtual labs provide unlimited access to class materials, thus facilitating better understanding. Virtual labs cannot replace hands-on training as yet, but the use of interactive simulations gives students an idea of what to expect in the real lab, thus cutting down the time spent learning new methods [7]. Virtual labs provide a risk-free learning environment, where individuals can learn from repeated practice, at their own pace, without using expensive resources or high maintenance equipment. Although virtual labs can link theoretical knowledge to practical applications in a lab set-up, these resources can't be expected to place individuals on an equal footing with those who have real-world training. Nonetheless, these platforms can be considered as a stage where students get a feel of what to expect in real-life experiments, acquire the necessary knowledge, and apply these concepts in physical labs at a future date. Virtual labs have already been shown to enhance student learning when used in conjunction with other teaching methods. Students are able to retain more information when visual and audio materials make classes more interactive and compel student participation.

Among the various virtual biotechnology labs in different stages of development, the HHMI Biomedical Interactive labs, MIT OCW, and the Virtual Labs Media Library at Summit, Stanford are the most popular (Texts S1 and S2). India's first comprehensive set of virtual labs are initiated through the “Sakshat” mission on higher education by the Ministry of Human Resource Development of the Government of India with an initial budget of nearly five billion Indian rupees. “Sakshat”— a Hindi word used to describe the witnessing of something that had previously only been imagined—is a content-delivery portal developed with an intention to portray India as a “knowledge super power” [8]. There are several emerging biotechnology virtual labs under this project covering different areas of modern biological sciences (Text S1). Virtual Proteomics Lab of Indian Institute of Technology (IIT) Bombay, one of the simulation-based virtual labs under this project, aims to share digital publication of high-quality research, college and university-level educational materials on proteomics, a newly emerging discipline of biotechnology, freely across the world. Open-source licensing of the course contents of the different biotechnology virtual labs under the “Sakshat” Labs Project is still under discussion; however, the education materials are freely accessible. A virtual proteomics lab is particularly valuable because, although proteomics has been incorporated in the academic curriculums of most of the universities and colleges around the world, only a few initiatives for proteomics virtualization exist.

## Virtualization in Proteomics: the Virtual Proteomics Laboratory at IIT-Bombay

With the goal of providing easily accessible, freely available and high-quality education across the globe, in February 2012 the Ministry of Human Resource Development (MHRD), Government of India launched our Virtual Proteomics Lab (VPL) as part of a comprehensive Virtual Lab Project, which is a collection of 91 virtual laboratories containing hundreds of experiments in nine disciplines of science and engineering [9]. Around 300 department heads, faculty, and staff representing over hundreds of eminent institutions across India have been convinced about the utility of both simulation-based and remote triggered Virtual Labs in routine course curriculum. This overall project is a mixture of static and remotely triggered labs in the engineering and science faculties that allow students to perform experiments in video simulations or use remotely triggered instruments. The Indian Institutes of Technology (IITs) and Amrita University played a cardinal role in development of virtual lab-based online educational courses in biotechnology. The biotechnology virtual labs under this project have a uniform appearance and follow a step-by-step method of explanation (see Text S2 for definitions of related terms).

**Figure 1:**
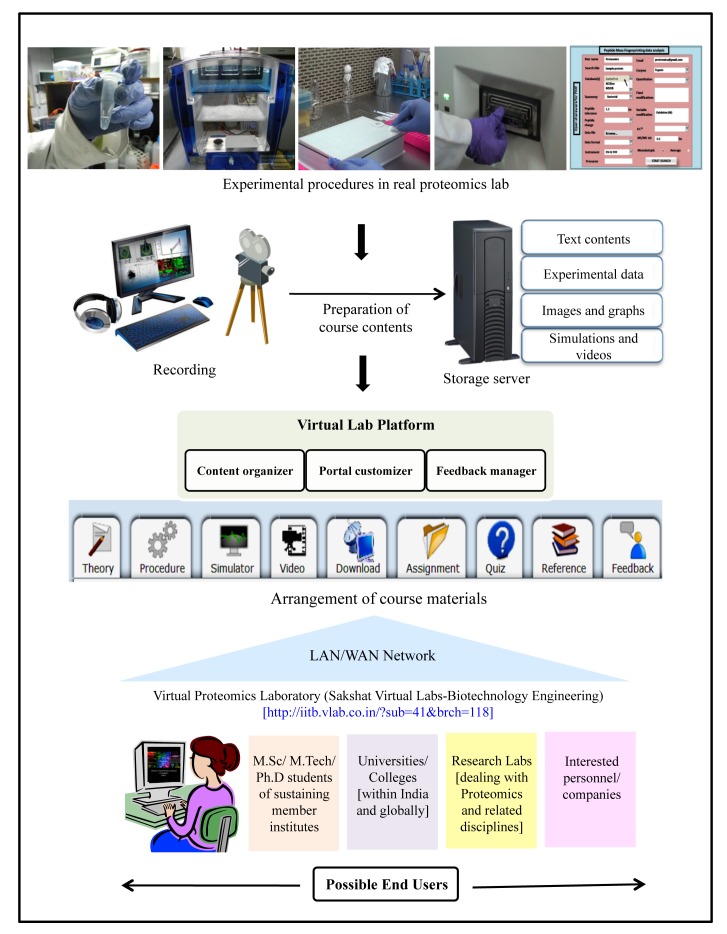
Schematic representation of the step-wise development of Virtual Proteomics Laboratory. Virtualization of proteomics data and experimental procedures from “bench-side” and storage in discrete compartments on the common “Virtual Lab server” for development of the course contents. Every experiment is explained in a step-by-step manner that follows the order of the tabs: theory, experimental procedure, simulator, video, download, assignment, quiz, references, and feedback. The server is connected to the Internet by a “LAN/WAN network” that contains the gateway portal and system firewall that protects the data from being changed by unauthorized people. The end users present on the “Client Side” can access the Virtual Lab via the LAN/WAN network portal. All course materials are freely available all over the world through the Internet and intended to be used in the teaching/learning experience particularly in universities and colleges. Moreover, the course content will be interesting and valuable for research labs working in the similar fields.

The VPL at IIT Bombay is part of this national project and solely dedicated to high-throughput proteome separation and analysis techniques, which are commonly used in basic and applied proteomics research (see Box 1). Already available at the URL http://iitb.vlab.co.in/?sub=41&brch=118, the lab consists of 12 experiments organized into three modules (Text S3). Students begin with protein separation through 2-D gel electrophoresis (2DE), identify the separated proteins using matrix-assisted laser desorption ionization time-of-flight mass spectrometry (MALDI-TOF MS) and analyze these proteins using bioinformatics techniques like sequence alignment, homology modeling, protein function annotation, and molecular docking. Every experiment has a sequentially arranged set of tabs that first takes a student through the theoretical and procedural aspects, followed by an interactive simulation and live experiment videos (Figure 1). The students can then test themselves using the self-assessment quizzes and assignments. All the written material and the assignments are available for download and references to additional reading materials are also provided.

**Box 1. Why Proteomics?**

Proteomics is one of the most flourishing areas of modern biotechnology research. The term proteome describes the protein complement expressed by a genome, while proteomics is the study of the entire compendium of proteins encoded by a genome for their expression, localization, interaction, and post-translational modifications. Over the last two decades development of several promising high-throughput proteomic technologies caused a phenomenal revolution in proteomics research and as a result this emerging platform has propelled its growth in multiple disciplines of life sciences [14],[15]. Direct involvement of proteins in most of the biological functions and a strong correlation of proteins with diseased conditions result into promising “bed-side” applications of clinical proteomics for early detection, rational therapeutic targeting, and patient-tailored therapy [16],[17]. Realizing the immense impact of proteomics on clinical and industrial research, universities and colleges have started integrating basic and applied proteomics as a part of their course curriculums to keep the students aware of the basic principles of different commonly employed proteomics technologies and provide hands-on training for some of the instruments. Although proteomics has been a prominent part of biomedical research for the last decade or so and has become an important part of biotechnology course work, establishment of several high-throughput technological facilities required for conducting proteomics courses in academic settings remains challenging because of the shrinking educational budgets, particularly in developing countries.

A few of the web-based open-learning repositories created over the last ten years have limited sections on Proteomics (Text S1). It is now apparent that virtual labs hold the promise of making the protocols for good quality practical coursework available to a much larger group of people than conventional on-campus education can achieve. Inclusion of virtual labs as a part of regular course curriculum will certainly be beneficial for developing countries like India where the number of qualified students is much larger than the number of universities able to afford the necessary infrastructures for advanced practical instruction. Thus, there is a specific need behind the development of virtual labs and other open-learning resources for proteomics and related subjects to spread the knowledge at university and college levels. To this end, development of our Virtual Lab in Proteomics at the Indian Institute of Technology (IIT) Bombay is a small endeavor to rectify this situation.

**Figure 2:**
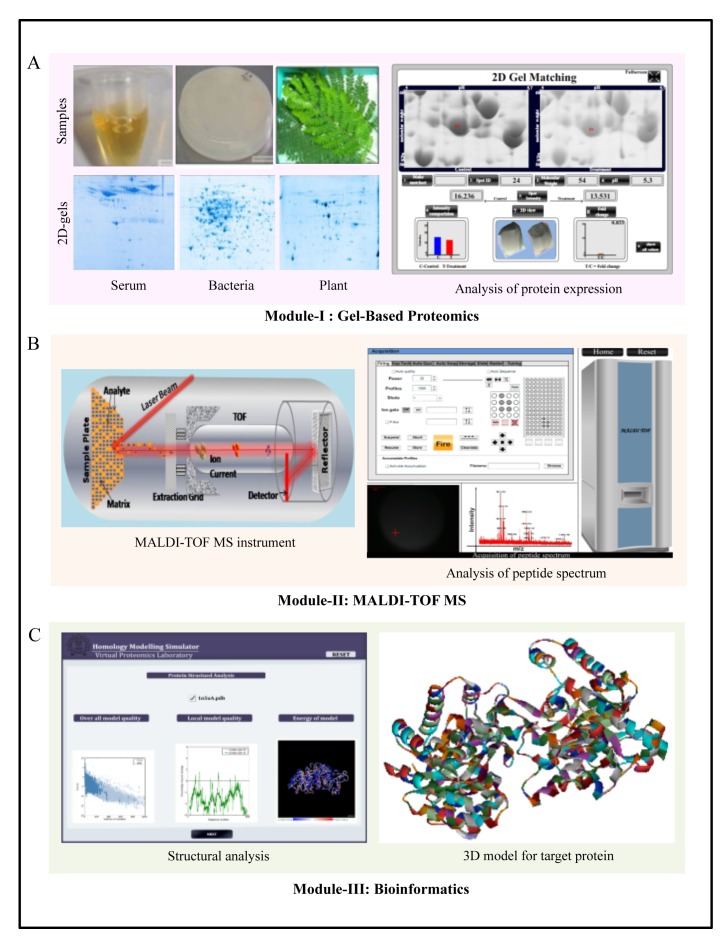
Organization of the course contents in the Virtual Proteomics Laboratory at IIT Bombay. (A) Module I (an overview of gel-based proteomics) consists of three experiments: gel-based proteomics (2DE) to analyze human serum, bacterial, and plant proteome; and analysis of differential expression of proteins between test and control samples. (B) Module II (an overview of MALDI-TOF MS) is focused on MS-based proteomics and consists of five experiments: in-gel digestion of proteins for MS analysis; sample preparation for the MALDI-TOF MS analysis; MALDI-TOF instrumentation and analysis of serum proteins; MS data analysis, peptide mass fingerprinting (PMF); and molecular weight determination of intact proteins. Schematic representation of MALDI-TOF instrument and operation procedure for generating peptide/protein spectrum for protein identification. (C) Module III (an overview of bioinformatics) covers the bioinformatics tools that are commonly used in proteomics. This module includes four experiments: sequence alignment, homology modeling, functional annotation, and molecular docking. Structural analysis and 3-D modeling of target proteins has been depicted.

Module I (gel-based proteomics) of this VPL consists of three experiments; each illustrating the isolation of proteins from different biological samples and their subsequent electrophoretic separation for differential proteome analysis: host responses against pathogenic infections using human blood serum, drug effects on bacteria using *Escherichia coli* cultures, and the consequences of stress on plants using plant leaf samples (Figure 2A). Using gel analysis software, students can compare the differences in the expression profiles of multiple proteins in the test and control samples to determine alterations in the proteome in response to external stimuli. Module II deals with the identification of these proteins using MALDI-TOF-MS. It consists of five experiments that take the students through all the steps of protein processing and preparation for mass spectrometric study. Students then learn the principles and operation of MALDI-TOF (Figure 2B) and how to use the peptide spectrum to identify proteins of interest with the help of software and databases. Module III (bioinformatics) is dedicated to four different bioinformatics techniques for protein analysis. Students first learn to look for homologous proteins using sequence alignment. This step is followed by structural prediction using homology modeling and functional annotation (Figure 2C) using available software and ontological databases, respectively. The molecular docking experiment teaches students the basics of protein–protein interactions and how these can be used to design ligands that can exert a specific effect on the target protein. The “do-it-yourself” assignments and self-assessment quizzes round up every experiment and allow students to evaluate themselves. All the material is available in pdf files and power point presentations for the experimental procedures and results are available. References are also provided for an in-depth study. There is a feedback section where students can leave their comments.

## Virtual Proteomics Labs of the Future

Our major aspiration behind creating this VPL has been to make a consolidated practical proteomics resource, where students can learn several important practical techniques in one program, because most of the programs are scattered among other online biology resources. It is a remarkable initiative for developing high-quality educational materials organized as courses for global distribution, originating outside of the USA or the EU. Since the materials associated with Virtual Labs Project of Government of India can be accessed freely through a common website: www.vlab.co.in, we expect the contents to be incorporated into the academic curriculum of different universities and colleges in India as per their requirements. The recent web site statistics—233,570 site visits and 1,034,443 page visits within just the last 6 months—and number of registered users—over 4,500 from 134 countries—certainly anticipate the forthcoming utility of this Virtual Labs Project at a global scale [9].

Course content of VPL has already been incorporated in the proteomics course curriculum of IIT Bombay, which is delivered to M.Sc./M.Tech./Ph.D. level students. The student response has been enthusiastic. Furthermore, quite a few educational and research institutes across India have shown interest in the course content and intend to introduce it as a part of their curriculum. The Virtual Lab project has been recognized in various publications recently, providing valuable platforms for disseminating the course contents to a wide variety of users at global level [10]–[12]. Though presently VPL covers only the most commonly used research techniques associated with gel and MS-based proteomics, we plan to expand its base to include protein microarrays and other advanced proteomics techniques, as well as a remotely triggered virtual proteomics laboratory. In the future, courseware could be individualized to fit every student's learning style and current knowledge base [13]. The material should preferably follow a predefined style that is readily understandable even to the beginners. We hope our endeavor can serve as a model for the future virtual proteomics labs and related subjects.

## Supporting Information

Text S1 **Some world leading virtual labs, e-learning, and open-learning resources for biotechnology and related disciplines.**
(DOC)

Text S2 **Virtual labs, definitions, and descriptions at a glance.**
(DOC)

Text S3 **Overview of Virtual Proteomics Laboratory at IIT Bombay.**
(DOC)

## References

[1] Atkins D. E, Brown J. S, Hammond A. L (2007). A review of the Open Educational Resources (OER) movement: achievements, challenges, and new opportunities.. http://www.hewlett.org/uploads/files/Hewlett_OER_report.pdf.

[2] OCW Consortium OpenCourseWare Consortium website.. http://ocwconsortium.org/.

[3] China Open Resources for Education (CORE) CORE official website.. http://www.core.org.cn.

[4] Kobayashi T, Kawafuchi A (2006). Japan Open Course Ware Consortium (JOCW): a case study in Open Educational Resources production and use in higher education.. http://www.oecd.org/dataoecd/61/2/37647892.pdf.

[5] Nilsson T (2003). Virtual laboratories in the life sciences. A new blueprint for reorganizing research at the European level.. EMBO Rep.

[6] Rumble G (1993). The economics of mass distance education.. Distance education: new perspectives.

[7] Scheckler R. A (2003). Virtual labs: a substitute for traditional labs?. Int J Dev Biol.

[8] National Mission on Education through Information and Communication Technology. Mission document.. http://sakshat.ac.in/PDF/Missiondocument.pdf.

[9] Press Information Bureau, Government of India (2012). Virtual labs project launched.. http://pib.nic.in/newsite/erelease.aspx?relid=80486.

[10] (2012). News: virtual labs for science education.. Nature India.

[11] Spinco Biotech (2012). E-learning and virtual labs.. Cutting Edge: Spinco Biotech Publication.

[12] Ray S, Koshy N. R, Reddy P. J, Srivastava S (2012). Virtual labs in proteomics: new E-learning tools.. J Proteomics.

[13] Diwakar S, Achuthan K, Nedungadi P, Nair B (2011). Enhanced facilitation of biotechnology education in developing nations via virtual labs: analysis, implementation and case-studies.. International Journal of Computer Theory and Engineering.

[14] Anderson N. L, Anderson N. G (1998). Proteome and proteomics: new technologies, new concepts, and new words.. Electrophoresis.

[15] James P (1997). Protein identification in the post-genome era: the rapid rise of proteomics.. Q Rev Biophys.

[16] Petricoin E. E, Paweletz C. P, Liotta L. A (2002). Clinical applications of proteomics: proteomic pattern diagnostics.. J Mammary Gland Biol Neoplasia.

[17] Petricoin E, Wulfkuhle J, Espina V, Liotta L. A (2004). Clinical proteomics: revolutionizing disease detection and patient tailoring therapy.. J Proteome Res.

