# Supplementary material for: Sakshat Labs: India's Virtual Proteomics Initiative
Source: PLoS Biol. 2012 Jul 10;10(7):e1001353. doi: 10.1371/journal.pbio.1001353 (PMC3393654; doi:10.1371/journal.pbio.1001353)
Supplement: Text S1 — Some world leading virtual labs, e-learning, and open-learning resources for biotechnology and related disciplines. (DOC) [file pbio.1001353.s001.doc]

| **Some world leading virtual labs, e-learning and open-learning resources for biotechnology and related disciplines**  **Text S1** | | | | |
| --- | --- | --- | --- | --- |
| **S. No** | **Virtual lab/**  **e-learning resource** | **Location** | **Subjects** | **URL** |
| 1. | Virtual Biology Labs at Rutgers | Rutgers University, New Jersey | Cell Biology, Plant Biology, Genetics | [http://bio.rutgers.edu](http://bio.rutgers.edu/) |
| 2. | Virtual Labs at SUMMIT - Stanford | Stanford University Medical Media and Information Technologies | Physiology, Biology, Immunology, Neuroscience, Health Education | <http://virtuallabs.stanford.edu/> |
| 3. | McGraw Hill Online Learning Center | McGraw Hill publishers | Basic Biology, Reproduction, Genetics, Ecology, Virology, Plant Biology, Animal Biology | <http://highered.mcgraw-hill.com/sites/0073031208/student_view0/virtual_labs.html> |
| 4. | Learn. Genetics | University of Utah | Basic Genetics, Stem Cells, Gene therapy, Cloning, Transgenics, Epigenetics, Genetic Technology | <http://learn.genetics.utah.edu/> |
| 5. | MIT Open Course Ware | Massachusetts Institute of Technology | Biochemistry, Genetics, Molecular Biology, Developmental Biology, Cell Biology, Stem Cells, System Biology, Neurology, Immunology, Health Science, Proteomics, Bioinformatics | <http://ocw.mit.edu/index.htm> |
| 6. | Serendip | Bryn Mawr College - Philadelphia | General Biology, Neurobiology, Evolution | <http://serendip.brynmawr.edu/serendip/> |
| 7. | HHMI Biomedical Interactive Virtual Labs | Harvard Hughes School of Medicine | Genetics, Cardiology, Neurophysiology, Microbiology, Immunology, Stem Cells, Genomics, Cancer, ELISA | [http://www.hhmi.org/biointeractive/vlabs](http://www.hhmi.org/biointeractive/vlabs/) |
| 8. | Virtual Laboratory – Colorado | Laboratory.net inc | Genetics, Fundamentals of Biology | <http://virtuallaboratory.colorado.edu/> |
| 9 | Biotechnology Virtual Labs | International Center For Agricultural Research in Dry Areas - Syrian Arab Republic | Farming related and scientific research tutorials | <http://www.icarda.org/Training_elearning.htm>  <http://learning.cgiar.org/moodle/> |
| 10. | National Program for Technology Enhanced Learning | Joint Collaboration of several Indian Universities | Biochemistry, Cell Biology, Molecular Biology, Fermentation Technology, Immunology, Proteomics, Microbiology, Biomathematics | <http://nptel.iitm.ac.in/courses.php?disciplineId=102> |
| 11. | “Sakshat” Virtual Biotechnology Engineering Labs* | Joint Collaboration of several Indian Universities | Neurophysiology, Biochemistry, Ecology, Immunology, Proteomics, Microbiology, Molecular Biology, Cell Biology, Instrumentation, Fermentation Technology, Cell Signaling | <http://www.vlab.co.in/ba_labs.php?id=6> |
| 12. | Department of Biology -Virtual Lab | Johns Hopkins University | Basic lab techniques, Instrumentation, Microscopy, Gel electrophoresis | <http://www.bio.jhu.edu/Undergrad/VirtualLabDemos.aspx> |
| 13. | **Cairo University, Faculty of Science E-learning facility** | Cairo University, Egypt | Biophysics, Botany , Zoology, Molecular Biology, Genetics | <http://elearning.cu.edu.eg/moodle/> |
| 14 | Annenberg Learner | Annenberg Foundation | Proteomics Theory and 2DE, Mass spectrometry, Protein Interactions and Microarrays | <http://www.learner.org/courses/biology/textbook/proteo/index.html> |
| 15. | Center for Cardiovascular Research - Molecular Biology | John A Burns School of Medicine, University of Hawaii - Center for Cardiovascular Research | SDS-PAGE, Western Blotting, ELISA, Protein microarrays | <http://ccrhawaii.org/index.php/protein-techniques> |

* **Major** **Biotechnology Virtual Labs under “Sakshat” project**

- **Biotechnology and Biomedical Engineering Labs - Amrita university**
  - These labs consist of a set of static and remotely triggered (RT) virtual labs with experiments related to Neurophysiology (RT), Biochemistry, Population Ecology, Immunology, Molecular Biology and Cell Biology.
  - Available at <http://amrita.vlab.co.in/?sub=3>
- **Biomedical and Signal Processing laboratory - College of Engineering, Pune**
  - This is a static virtual lab that explains the applications of several instruments used in cardiac care such as the pacemaker and defibrillator using animated simulations.
  - Available at <http://coep.vlab.co.in/?sub=25>
- **Bioreactor Modeling and Simulation Lab - Indian Institute of Technology Delhi**
  - The Bioreactor Virtual Lab at IIT Delhi is a static lab with modules covering the basics of bioreactors and fermentation technology with the help of animated simulations.
  - Available at <http://iitd.vlab.co.in/?sub=63>
- **Virtual Proteomics Laboratory - Indian Institute of Technology Bombay**
  - The proteomics Virtual Lab at IIT Bombay is a static Virtual Lab dedicated to important, high-throughput proteome separation and analysis techniques and their applications in fundamental and clinical research.
  - Available at <http://iitb.vlab.co.in/?sub=41&brch=118>
