# Supplementary material for: Sakshat Labs: India's Virtual Proteomics Initiative
Source: PLoS Biol. 2012 Jul 10;10(7):e1001353. doi: 10.1371/journal.pbio.1001353 (PMC3393654; doi:10.1371/journal.pbio.1001353)
Supplement: Text S2 — Virtual labs, definitions, and descriptions at a glance. (DOC) [file pbio.1001353.s002.doc]

**Text S2**

**Virtual Labs, definitions and descriptions at a glance**

**1.** **HHMI Biomedical Interactive labs**- The Virtual Lab series produced by the Howard Hughes Medical Institute are fully interactive biomedical laboratory simulations that include a bacterial identification lab, a cardiology lab, a neurophysiology lab and a virtual ELISA using human antibodies to diagnose disease [S1]**.**

**2. Learn. Genetics- University of Utah** - This series delivers educational materials on genetics, bioscience and health topics designed for teachers, students and members of the public organizations. They also provide Virtual Labs for stem cell differentiation and gene therapy [S2].

**3. MIT-OCW:** Open Course Ware - e-learning project of Massachusetts Institute of Technology that has generated freely available web versions of all courses currently taught at MIT [S3, S4].

**4. NME-ICT:** The National Mission for Education through Information and Communication technology, started by the Indian MHRD in 2009; this web-based learning program has sponsored the development of India's Sakshat Virtual Labs [S5].

**5. NPTEL:** National Program on Technology Enhanced Learning - India's e-learning initiative started by the Ministry of Human Resource Development (MHRD) in 2003 to introduce web-based learning to the Engineering and other Science streams through freely available web-based lectures and video courses. It offers 34 Biotechnology courses currently [S6].

**6. Open Course Ware (OCW):** Educational resources developed by various universities, freely available all over the world through the Internet and intended to be used in the teaching/learning experience.

**7**. **Open Educational Resources (OER):** Educational resources including full courses, course materials, modules, textbooks, streaming videos, tests, software, pedagogical materials, games, simulations and any other tools, materials, or techniques released under an intellectual property license that makes the course contents freely accessible and re-useable for teaching, learning, research purpose by anyone, anytime through Internet (modified from the William and Flora Hewlett Foundation [S7]).

**8. Sakshat Virtual Labs:** India's foremost comprehensive set of virtual labs under the NME-ICT initiative of the Ministry of Human Resource Development (MHRD) involving several institutes and universities from all over India [S8].

**9. Static Virtual lab** - A virtual lab where students can perform experiments using interactive simulations that mimics the real time lab experiment in a pre-defined controlled environment.

**10. Remotely Triggered Virtual Lab** - A virtual lab where students can perform experiments by controlling actual instruments in a physical lab using a web-based program.

**11. Virtual Labs at SUMMIT** – Stanford - started in 1998 with funding from the Howard Hughes Medical Institute; these labs consist of interactive tutorials in biology, medicine, health and humanities [S9].

**Supplemental References**

**S1.** HHMI Virtual labs-Home page. Available: <http://www.hhmi.org/biointeractive/vlabs/>. Accessed 24 April 2012.

**S2.** Learn. Genetics-About Us. Available: <http://learn.genetics.utah.edu/gslc/>. Accessed 24 April 2012.

**S3.** MIT-OCW History. Available: <http://ocw.mit.edu/about/our-history/>. Accessed 24 April 2012.

**S4.** MIT OCW Site statistics. Available: http://ocw.mit.edu/about/site-statistics/. Accessed 24 April 2012.

**S5.** National Mission on Education through Information and Communication Technology- Mission Document. Available: <http://sakshat.ac.in/PDF/Missiondocument.pdf>. Accessed 24 April 2012.

**S6.** National Programme on Technology Enhanced learning-Project Document. Available: <http://nptel.iitm.ac.in/index.php>. Accessed 24 April 2012.

**S7.** Atkins DE, Brown JS, Hammond AL (2007) A Review of the Open Educational Resources (OER) movement: achievements, challenges, and new opportunities. Available: <http://www.hewlett.org/uploads/files/Hewlett_OER_report.pdf>. Accessed 24 April 2012.

**S8.** Sakshat virtual labs website. Available: http://www.vlab.co.in/ba_labs.php?id=6. Accessed 24 April 2012.

**S9.** Virtual labs media library-Homepage- SUMMIT, Stanford. Available: http://virtuallabs.stanford.edu/. Accessed 24 April 2012.
