# Supplementary material for: Sakshat Labs: India's Virtual Proteomics Initiative
Source: PLoS Biol. 2012 Jul 10;10(7):e1001353. doi: 10.1371/journal.pbio.1001353 (PMC3393654; doi:10.1371/journal.pbio.1001353)
Supplement: Text S3 — Overview of Virtual Proteomics Laboratory at IIT Bombay. (DOC) [file pbio.1001353.s003.doc]

[
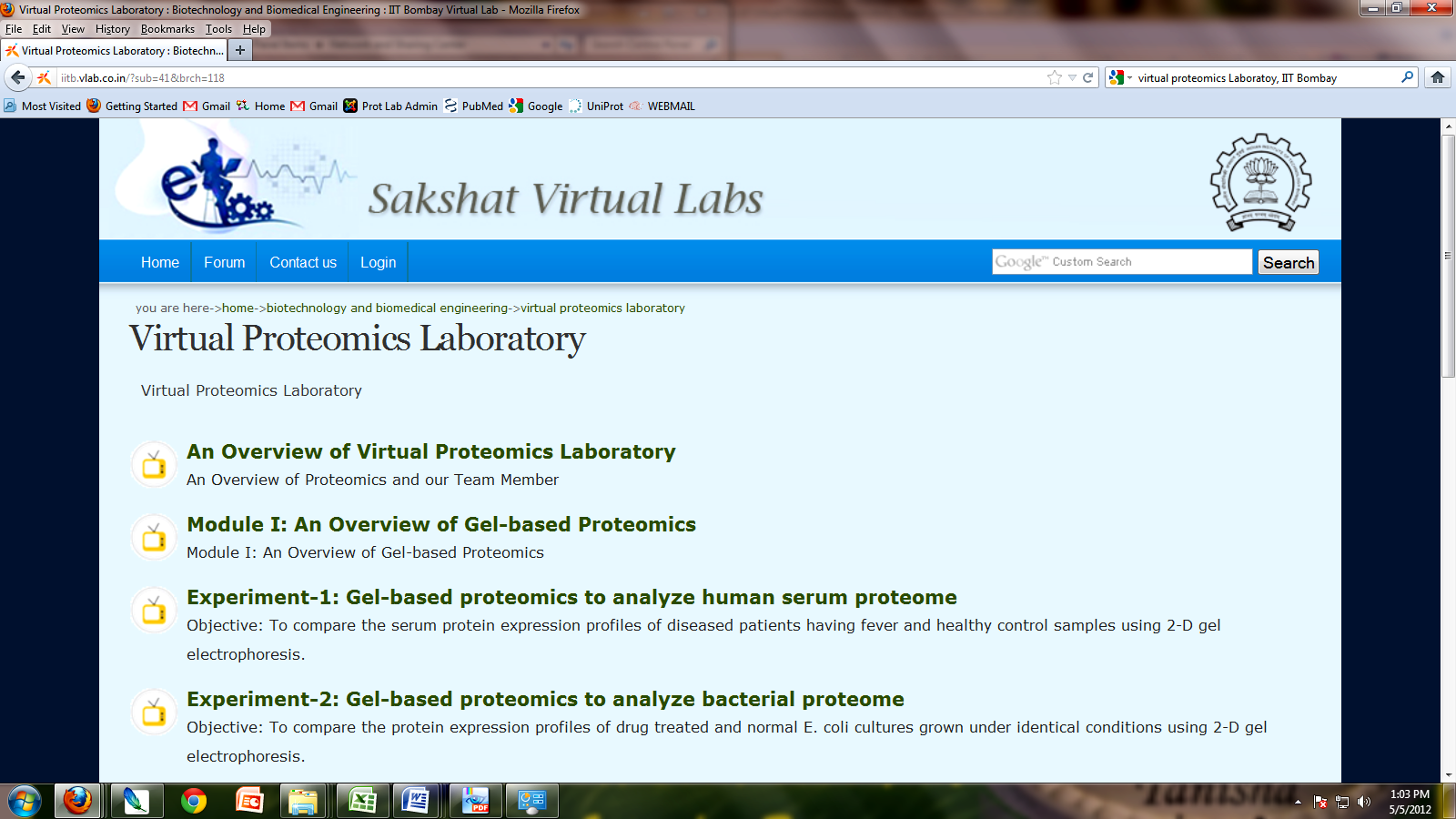
**An Overview of Virtual Proteomics Laboratory**](http://iitb.vlab.co.in/?sub=41&brch=118&sim=372&cnt=324) **at IIT Bombay**

**Module I: An Overview of Gel-based Proteomics**

**Experiment-1: Gel-based proteomics to analyze human serum proteome**

Objective: To compare the serum protein expression profiles of diseased patients having fever and healthy control samples using 2D gel electrophoresis

**Experiment-2: Gel-based proteomics to analyze bacterial proteome**

Objective: To compare the protein expression profiles of drug treated and normal *E. coli* cultures grown under identical conditions using 2D gel electrophoresis

**Experiment-3: Gel-based proteomics to analyze plant proteome**

Objective: To compare the protein expression profiles of plant leaves grown under conditions of drought (stress) and normal conditions using 2D gel electrophoresis

**Text S3**

**Module II: An Overview of MALDI-TOF MS**

**Experiment-4: In-gel digestion of proteins for MS analysis**

Objective: To excise the spots from 2D or 1D SDS-PAGE gels and perform in-gel trypsin digestion

**Experiment-5: Sample preparation for the MALDI-TOF MS analysis**

Objective: To prepare the test and standard samples for the MALDI-TOF MS analysis

**Experiment-6: MALDI-TOF instrumentation and analysis of serum proteins**

Objective: MALDI-TOF instrumentation and generation of peptide mass fingerprinting (PMF) from the serum proteins

**Experiment-7: MS data analysis - Peptide Mass Fingerprinting (PMF)**

Objective: To analyze the MALDI-TOF peptide mass fingerprinting data

**Experiment-8: Molecular Weight Determination of Intact Protein Using MALDI-TOF**

Objective: To determine the molecular weight of intact protein using MALDI-TOF

**Module III: An Overview of Bioinformatics**

**Experiment-9: Sequence Alignment**

Objective: To perform sequence alignment

**Experiment-10: Homology Modelling**

Objective: To perform homology modelling

**Experiment-11: Protein Function Annotation**

Objective: To perform protein function annotation

**Experiment-12: Molecular Docking**

Objective: To perform molecular docking for studying molecular interactions
